# Supplementary material for: Djhsp60 Is Required for Planarian Regeneration and Homeostasis
Source: Biomolecules. 2022 Jun 9;12(6):808. doi: 10.3390/biom12060808 (PMC9221281; doi:10.3390/biom12060808)
Supplement: Supplementary file 1 [file biomolecules-12-00808-s001.zip › Supplementary figures and figure legends version2.pdf]

Supplementary figures and figure legends

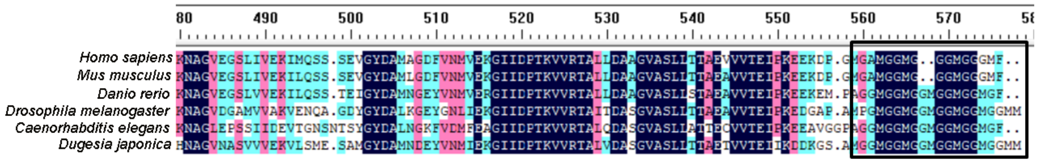

Figure S1. Multiple alignment of DjHSP60 with other HSP60 proteins from different species. The conserved GGM repeats at C-terminal shared by mitochondrial HSP60 family were boxed.

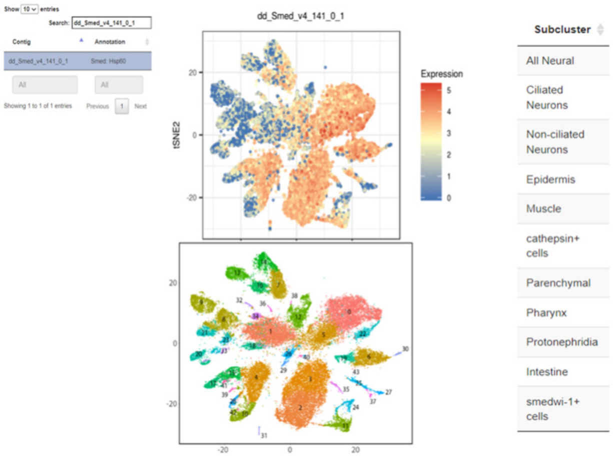

Figure S2. Expression patterns of hsp60 transcripts revealed by single cell data analysis.

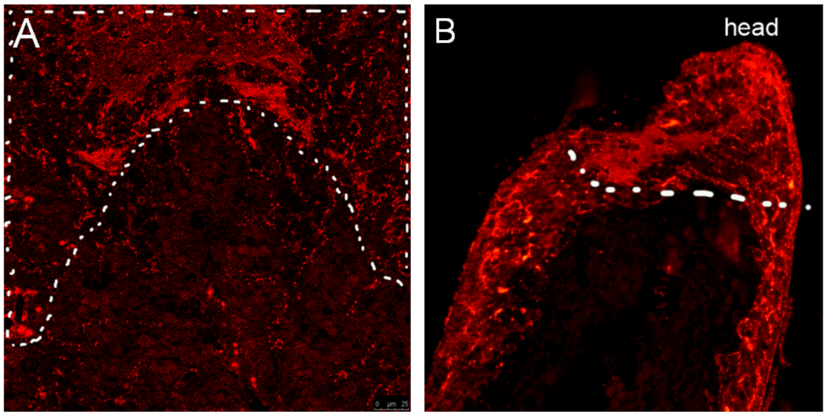

Figure S3. Head region of planarian revealed by antibody of HSP60 staining. The numbers of red dots (HSP60 protein) in head neural region are much more than in other region. (A) Horizontal section. Inverted “U” shape of brain structure was circled by white line (B) Sagittal section.

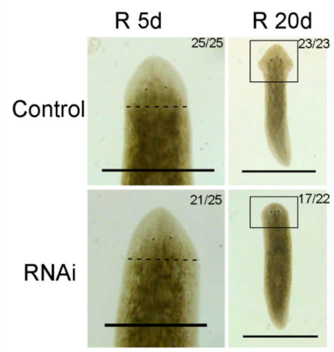

Figure S4. Effects of RNAi on planarian regeneration. After 14 days of feeding dsRNA, animals without head-defect were cut for regeneration. After 5 days of regeneration, most of RNAi-animals can regenerate normal heads. After 20 days of regeneration, the newly regenerated RNAi-animals showed the loss of auricles, and the head became round. The broken black line represents the border between blastema and old tissue. The difference between control and RNAi was boxed. Scale bar: 1 mm.

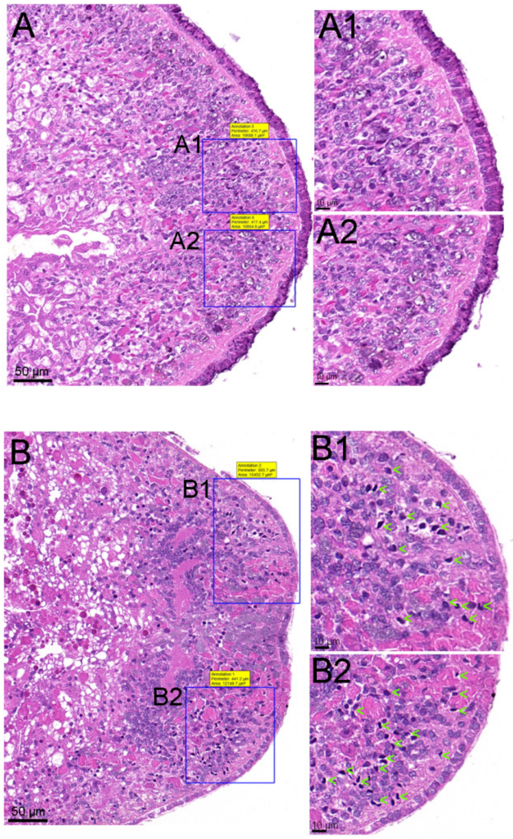

Figure S5. The cytomorphological difference of head region between the control and RNAi revealed by HE staining. (A) Control sample. (B) RNAi sample. Green arrowheads indicate the dead cells. No typical dead cells can be seen in control sample. Animals with mild head-defect were used for this experiment.

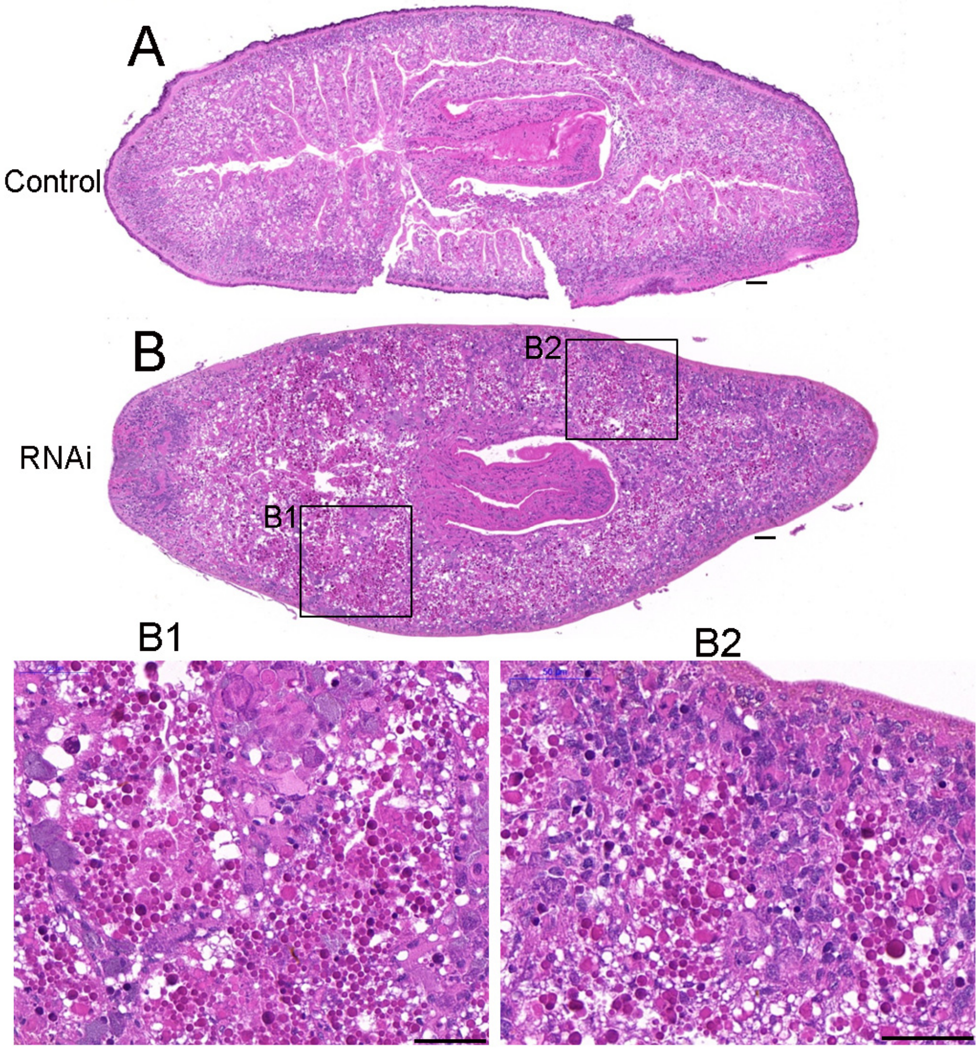

Figure S6. Morphological changes of intestinal tissues after RNAi revealed by HE staining. Scale bar: 50 μm.

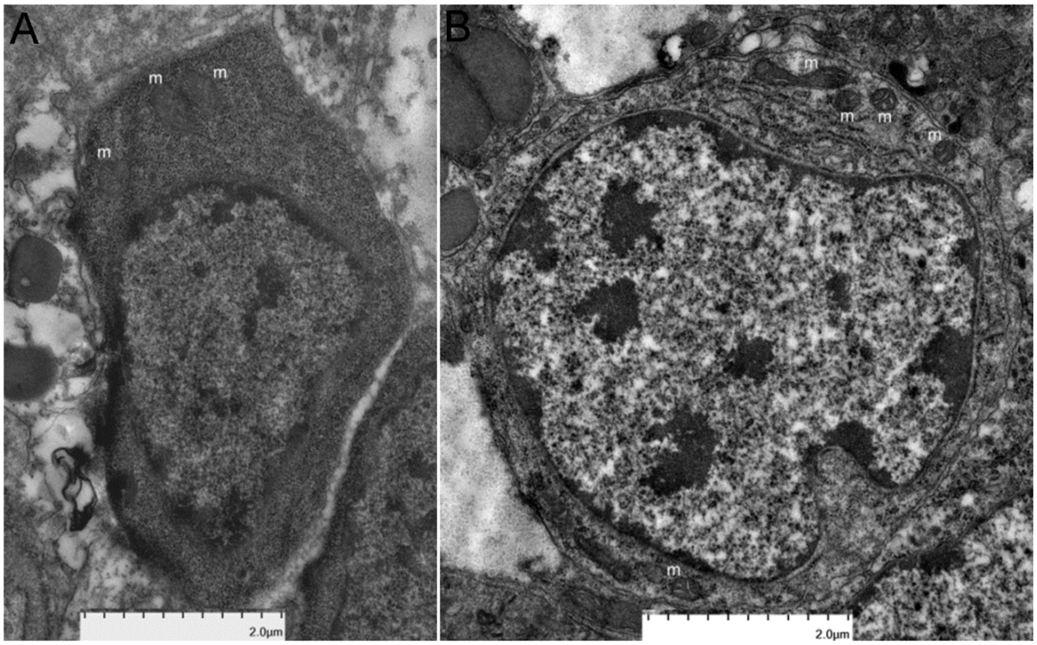

Figure S7. Neoblast-like cells in planarian revealed by TEM. The morphological characteristics of neoblasts: they are small in size, have a large nucleus and scant cytoplasm with a few organelles, including free ribosomes, mitochondria and chromatoid bodies. (A) a neoblast-like cell. (B) an early neoblast progeny like cell. m: mitochondria.

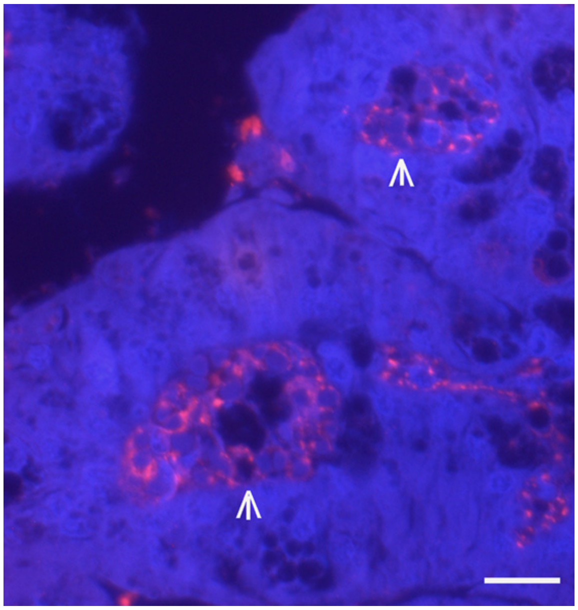

Figure S8. Local location of neoblasts-like cells. At this region, HSP60-positive cells (Red) are supposed to be neoblasts-like cells, which are surrounded by intestinal cells. Fluorescent-immunostaining section detected by Flur 594. Scale bar: 20 μm.

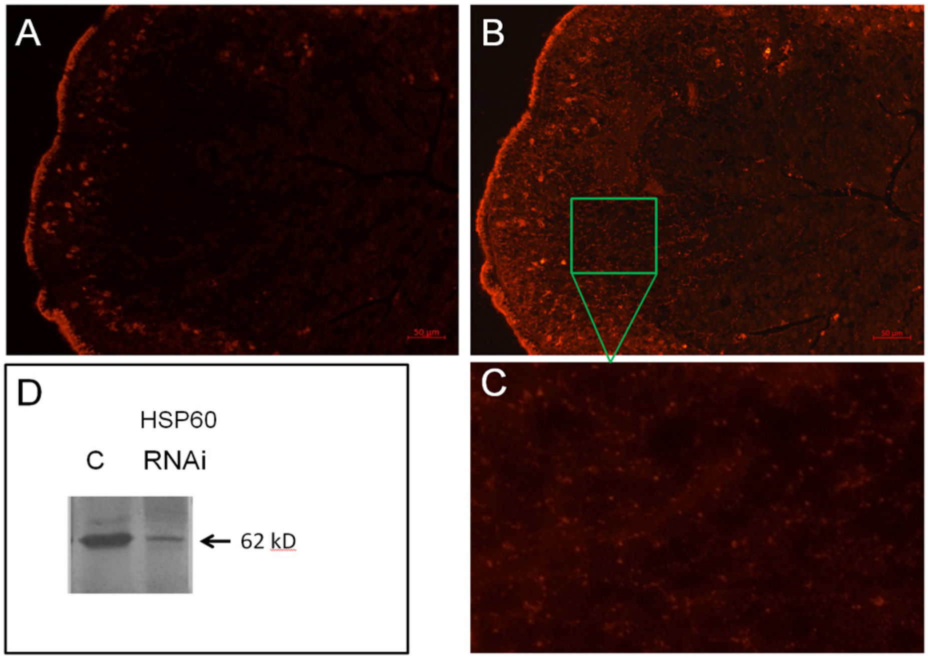

Figure S9. Specificity of the commercial HSP60 antibody revealed by fluorescent-immunostaining and Western blot. **(A)** PBST instead of primary antibody of HSP60 was used as negative control. Except for non-specific staining, no specific staining can be seen. **(B)** Distribution of HSP60 protein in planarian head region. Compared with the negative control, the positive signals are very evident, and the strong positive signals are located at brain region. **(C)** Magnification of the boxed region in B. Positive signals are red dots, which are very different from non-specific signals. **(D)** Specificity of the commercial HSP60 antibody was detected by western blot. 20 μg protein was loaded in the gel.
